# Impact of refrigeration of different Resin composite restorative materials on the marginal adaptation in class II restorations

**DOI:** 10.1186/s12903-024-04886-3

**Published:** 2024-10-03

**Authors:** Omar Abd El-Maksoud, Hamdi Hamama, Ramy Ahmed Wafaie, Noha El-Wassefy, Salah Hasab Mahmoud

**Affiliations:** 1 Conservative Dentistry Department, Faculty of Oral and Dental Medicine, Delta University for Science and Technology, Gamasa, Egypt; 2 Conservative Dentistry Department, Faculty of Dentistry, Mansoura University, Mansoura, Egypt; 3 Dental Biomaterials Department, Faculty of Dentistry, Mansoura University, Mansoura, Egypt; 4 Faculty of Dentistry, Mansoura National University, Gamasa, Egypt

**Keywords:** Class II restorations, Marginal adaptation, Methacrylate composite, Ormocer, Storage temperature

## Abstract

**Background:**

The pre-polymerization temperature of resin composite restorative materials could influence their adaptation to cavity details. As a current debate is existing about the refrigeration of resin composite restorative materials, this study was designed to assess the effect of refrigeration of 3 types of resin composite restorative materials with different matrix systems on their marginal adaptation in Class II restorations.

**Methods:**

Forty-two sound maxillary molars, each with two separated Class II cavities, were used in this study. The teeth were assigned into 3 main groups (*n* = 14) according to the restorative /adhesive system used; an Ormocer-based composite (Admira Fusion/Futurabond M+, Voco GmbH, Cuxhaven, Germany), a methacrylate modified Ormocer-based (Ceram.X SphereTEC One/Prime&Bond Universal, Dentsply Sirona GmbH Konstanz, Germany), and a methacrylate-based (Tetric N-Ceram/Tetric N-Bond Universal, Ivoclar Vivadent AG, Schaan, Liechtenstein). Each group was then divided into 2 subgroups (*n* = 14) according to the gingival margin location; 1 mm above and 1 mm below the cemento-enamel junction (C.E.J). Each subgroup was further divided into 2 categories (*n* = 7) according to the storage temperature; stored at room temperature or stored in refrigerator at 4°- 5° C. Epoxy resin replicas were observed under scanning electron microscope (SEM) to examine the marginal gaps. A gab scoring system was used to assess the marginal adaptation of each restoration by giving scores on the basis of measurements of the maximum marginal gaps. The data obtained were statistically analyzed using the Chi-square test at a significance level of *p* < 0.05.

**Results:**

None of the tested groups exhibited 100% gap-free margins irrespective of margin location or storage temperature. For both storage temperatures, no statistically significant difference was observed among all tested groups either with margins located above or below C.E.J (*p* > 0.05). As well, there was no statistically significant difference when comparing both marginal locations for each material (*p* > 0.05). Regarding the effect of storage temperature, statistically significant difference was only observed between the room-temperature stored groups with margins located above C.E.J and their corresponding groups stored in refrigerator (*p* < 0.05).

**Conclusion:**

The refrigeration of resin composite restorative materials prior to the restorative procedures revealed a deleterious effect on marginal adaptation of the restorations with margins located in enamel regardless the type of material used.

## Background

Despite the long service history of dental amalgam as the most commonly used restorative material, its use has significantly decreased in many highly developed countries [1]. This is mainly attributed to the noticeable demand for esthetic tooth-colored restorations, the shift towards the minimally invasive approach in addition to the systemic and environmental harmful effects through mercury exposure [2]. Moreover, the Minamata convention called for a gradual phase-down of dental amalgam through greater affirmation on prevention and searching for new dental alternative materials [3]. Following these recommendations, the usage of resin composite restorative material has been adopted as a directly-placed substitute for dental amalgam [4].

Resin composite restorative materials have possessed several advantages such as tooth-color matching through various shades and translucencies securing high patients’ satisfaction [5], possibility of repairing defective restorations, adhesion to both enamel and dentin using adhesive agents without the need for extensive retention means or undesirable sacrifice of sound tooth structure thus reinforcing the remaining tooth structure and achieving acceptable prognosis [6, 7].

Since the earlier introduction of Bis-GMA (a bisphenol-A glycidyl methacrylate)-based resin composite restorative materials [8], they were only applied in shallow/small cavities. The inherent polymerization shrinkage was considered one of the most serious drawbacks limiting their usage [9]. The generated stresses associated with the polymerization shrinkage were responsible for loss of marginal integrity [10], gap formation [11], interface fracture, and micro-leakage [12]. In attempts to overcome these shortcomings, several strategies have been introduced such as using incremental placement technique, soft start polymerization protocol, stress absorbing cavity liners, as well as modifying the material composition with a focus on the filler system enhancements [13]. Recently, greater attention was directed to enhance the resin matrix formulations [14], by developing new monomers with the aim of introducing low-shrink composite systems [10].

Ormocer, the acronym for organically modified ceramic, was synthesized through a solution and gelation process (sol-gel process) [15]. The siloxane oligomeric nanostructure is formed by hydrolysis and polycondensation of functionalized organosilanes groups [16]. This results in a matrix of long inorganic silica chain backbones with organic polymerizable side chains, able to be cured through light-induced polymerization [17]. The resultant oligomers can substitute the classic resinous monomers and a complex network of three dimensions is constructed by these functional groups polymerization [18].

One of the requisites of durable restorative material is to adapt properly and seal the cavity walls for assuring long-lasting performance [19]. Independent of the bonding efficiency of the used adhesive system, achieving a perfect marginal seal is very challenging [20]. Poor marginal adaptation may lead to leakage of oral fluids through tooth-restoration interface which results in postoperative sensitivity, marginal staining [21], and secondary caries that is often considered one of the main reasons for restoration failure [22].

The pre-cure temperature is considered an influential factor that affects the polymerization kinetics and hence, the features of the resultant polymer [23]. A temperature between 4 °C and 20 °C is recommended for storing resin composite syringes to obtain maximum efficacy [24]. Most dental practitioners store resin composite restorative materials in the refrigerators in order to extend their shelf life. Some studies [25, 26] revealed that refrigerating the resin composite restorative materials had no adverse effects on its properties. On the contrary, other reports [27, 28] have found that such behavior may result in undesirable consequences. A dissension exists around the suitability and the impact of storing resin composite restorative materials at low temperatures. Therefore, the aim of this study was to assess the effect of refrigeration of three resin composite restorative materials with different matrix systems on their marginal adaptation in Class II restorations.

## Methods

### Restorative materials

The current study was conducted using three commercially-available resin composite restorative materials with their corresponding adhesive systems as follows; an Ormocer-based composite (Admira Fusion/Futurabond M+, Voco GmbH, Cuxhaven, Germany), a methacrylate modified Ormocer-based (Ceram.X SphereTEC One/Prime&Bond Universal, Dentsply Sirona GmbH, Konstanz, Germany), and a methacrylate-based (Tetric N-Ceram/Tetric N-Bond Universal, Ivoclar Vivadent AG, Schaan, Liechtenstein). The shade of the three resin composite restorative materials used was uniformed to A2 shade. Each restorative system was used according to manufacturers’ instructions. The full description of all materials is summarized in Table 1. The curing of the resin composite restorative materials was performed using a well-controlled light-emitting diode (LED) Elipar S10 (3 M Oral Care, St. Paul, MN, USA) light-curing device with a wavelength between 430 and 480 nm and a light intensity of 1200 mW/cm^2^ as measured by a dental radiometer (Bluephase, Ivoclar Vivadent AG) [29].

**Table 1:** Materials used in the study

Materials	Type	Manufacturer	Composition			Batch No.
Admira Fusion	Ormocer-based composite	Voco GmbH, Cuxhaven, Germany	Matrix	Filler	Filler degree	1,939,483
			Ormocer	silicon oxide nanofiller, glass-ceramic filler	84% by wt.	
Futurabond M+	Universal adhesive		HEMA, Bis-GMA, acidic adhesive phosphate monomer, ethanol, catalyst			2,113,221
Ceram.X SphereTEC One	Methacrylate modified Ormocer-based composite	Dentsply Sirona GmbH, Konstanz, Germany	Methacrylate-modified polysiloxane, Poly-urethane methacrylate, Bis-EMA, TEGDMA	prepolymerized spherical fillers, Barium-aluminum borosilicate glass, ytterbium fluoride, methacrylate functionalized silicon dioxide nanofiller	77–79% by wt.	1,908,000,044
Prime&bond universal	Universal adhesive		Multifunctional acrylate, bifunctional acrylate, PENTA, MDP, isopropanol, water, initiator			2,002,000,692
Tetric N Ceram	Methacrylate -based composite	Ivoclar Vivadent AG, Schaan, Liechtenstein	UDMA, Bis-GMA, Ethoxylated Bis-EMA, TEGDMA	barium glass, ytterbium trifluoride, mixed oxide, silicon dioxide prepolymers	80–81% by wt.	X49739
Tetric N Universal Bond	Universal adhesive		BisGMA, HEMA, MDP, MCAP, D3MA, water, ethanol			X43844

### Teeth selection

Forty-two sound freshly extracted maxillary molars were collected from healthy individuals from the Department of Oral Surgery, Faculty of Dentistry, Mansoura University, Mansoura, Egypt following the International/Faculty infection control guidelines (ethical approval No. A09030320). To remove the soft tissue remnants, a hand scaler (Zeffiro, Lascod, Florence, Italy) was used. For disinfection, the teeth were stored in a disinfectant solution (0.5% chloramine-T) for 72 h [30]. Rubber cup and pumice water slurry were used to clean the teeth. All the collected teeth were free from cracks as determined by examination under 30x magnification using a binocular stereomicroscope (SZ TP, Olympus, Tokyo, Japan). The crown dimensions of these selected teeth were as follows; 10.0 to 11.0 mm buccolingual diameter, 9.0 to 10.0 mm mesiodistal diameter, and 7.0 to 7.5 mm cervical-occlusal height. Dimensions were measured with the aid of a digital caliper. The teeth were then stored in distilled water at 37 °C ± 1 °C using an incubator (BTC, BioTech Company, Cairo, Egypt). Teeth were removed only during the test procedures in order to avoid dehydration [31]. Teeth were fixed using in auto polymerizing acrylic resin (Acrostone, Cairo, Egypt) having their roots held up to 2 mm below C.E.J [32].

### Study design

Teeth were assigned mainly into 3 groups (*n* = 14) according to the resin composite restorative/adhesive system used. Each group was then divided into 2 subgroups (*n* = 14) according to the gingival margin location; 1 mm above and 1 mm below the cemento-enamel junction (C.E.J). Each subgroup was further divided into 2 categories (*n* = 7) according to the storage temperature; stored at room temperature or stored in refrigerator at 4°- 5° C.

### Specimen preparation

Two separated proximal Class II preparations (occluso-mesial and occluso-distal) were prepared in each selected tooth using a straight fissure diamond instrument (6836 Kr 314 018; Komet, Brasseler, Lemgo, Germany) in a high-speed handpiece (Sirona T3, Benshim, Germany) under copious air-water cooling. To ensure the efficacy of cutting, the rotary cutting instrument was replaced after every five preparations. Standardization of the cavity preparation was performed by fixing the handpiece in specially designed jig and fixer fabricated in the Department of Mechanical Design and Production Engineering, Faculty of Engineering, Mansoura, Mansoura University [33].

**Fig. 1:**
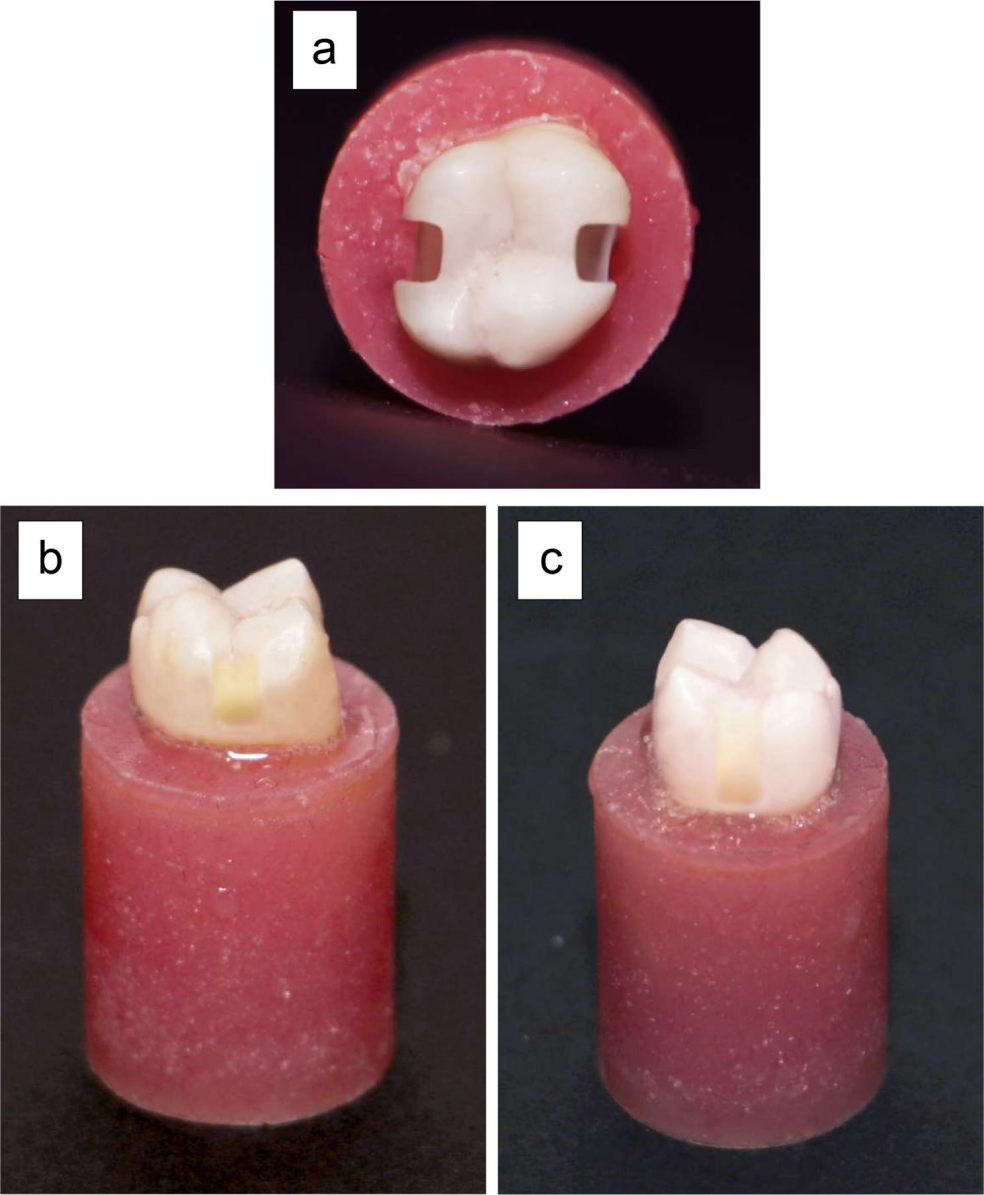
A representative photograph showing: **a**: occlusal view of maxillary molar with two separated proximal Class II cavity preparations, **b**: prepared proximal box with margin located 1 mm above C.E.J, **c**: prepared proximal box with margin located 1 mm below C.E.J

The buccolingual width of proximal boxes was 1/3 of the intercuspal distance. The axial wall of each box was at 2 mm depth from each proximal surface. The proximal gingival margin was placed in enamel, 1 mm above C.E.J in one proximal side, and the other margin was located in cementum, 1 mm below C.E.J [34]. Each box had parallel buccal and lingual walls, with a 90-degree cavosurface angle and rounded internal angles (Fig. 1). The dimensions of each prepared cavity were measured using digital caliper [33].

### Restorative procedures

A metal matrix was used to reestablish the proximal contours. Following selective enamel etching technique, all enamel margins were etched with a 37% phosphoric acid etching gel (Scotchbond, 3 M Oral Care) for 15–30 s then rinsed thoroughly with a vigorous stream of water for 5 s and dried with oil-free air. Whilst, all adhesive systems were employed in self-etch mode for cementum margins without a separate etching step. Each adhesive was applied and scrubbed into the cavity surface for 20 s using a micro-brush tip and gently dried by oil-free air for 2–5 s following the manufacturers’ recommendations. Light curing was then performed for 10 s. Each material was applied incrementally (up to 2 mm thickness), adapted to the cavity surface using modeling instrument (OptraSculpt, Ivoclar Vivadent AG), and light-polymerized from the occlusal aspect for 20 s. To ensure maximum curing efficiency, both proximal surfaces were cured after matrix removal. Regarding refrigerated groups, syringes were stored in refrigerator for at least 30 min in order to stabilize the temperature of 4°-5 °C. The syringe was removed from the refrigerator and the material was immediately applied to the cavity before its temperature changed appreciably. The composite syringe was returned to the refrigerator and replaced by another refrigerated one to make another specimen.

**Fig. 2:**
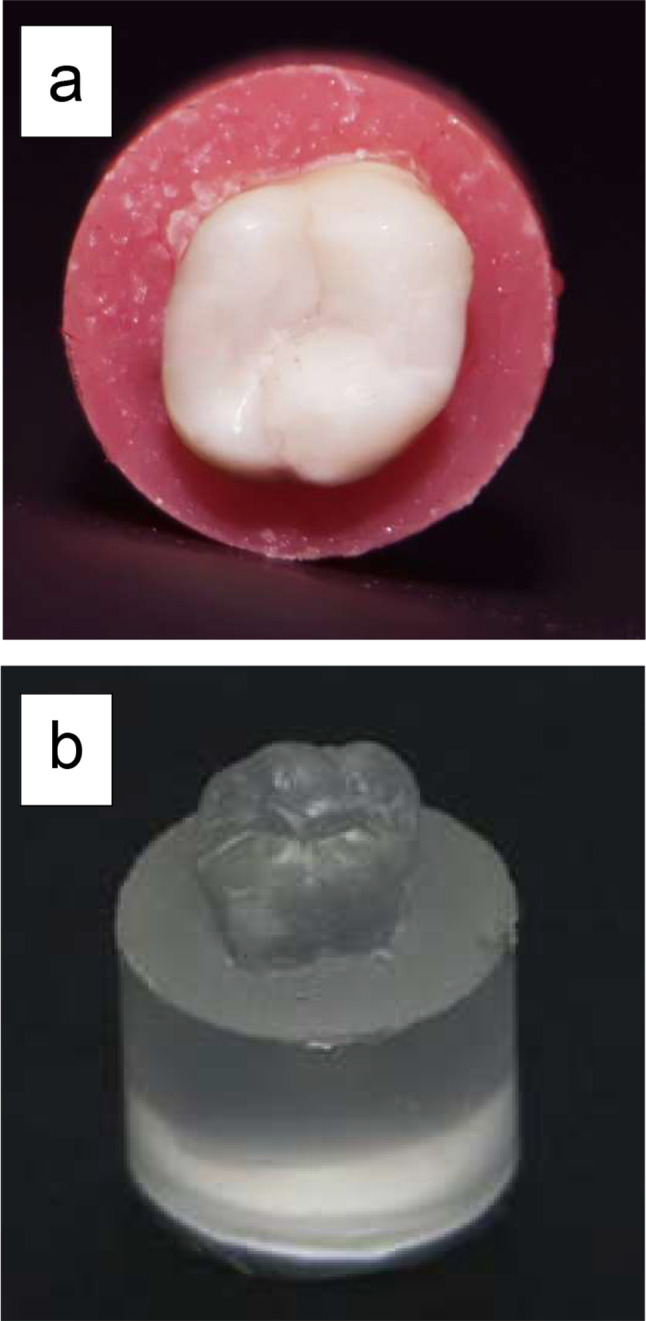
A representative photograph showing: **a**: finished and polished composite restoration, **b**: positive epoxy resin replica

Finishing of the restorations was performed using high-speed diamond finishing instruments (4092.314, Komet) under copious air-water cooling. Proximal overhangs were removed using a No. 12 scalpel blade mounted in Bard-Parker handle with light pressure. The proximal surface was finished and polished with flexible discs (Sof-Lex XT Pop On, 3 M Oral Care) according to the recommended sequence (coarse, medium, fine and superfine). To achieve a smooth surface, flexible points and aluminum oxide impregnated cups (Enhance, Dentsply Caulk) were used (Fig. 2-a) [35, 36]. Specimens were stored in distilled water for 24 h prior to impression making.

### Testing

Using one-step putty-wash technique, impression of each specimen was taken by polyvinyl-siloxane impression material (Elite HD + putty and light body, Zhermack SpA, Badia Polesine, Italy). The light body impression material was applied to the restored tooth by an auto-mixing gun. The base and the catalyst of the putty impression material were mixed in equal quantities and then packed into a custom-made plastic ring that coated with adhesive (Coltene Adhesive AC, Coltene/Whaledent AG, Altstätten, Switzerland). The ring was then held in a parallel direction to the tooth-long axis without pressure until the impression material was set [32].

The impressions were poured with epoxy resin (SwissChem, 6th of October City, Giza, Egypt). The positive replicas were then removed from the impressions after setting at room temperature for 24 h (Fig. 2-b). After trimming, all replicas were held on standard aluminum stubs using a carbon conductive double-sided adhesive tape. The replicas were then sputter-coated with gold (SPI-Module TM Sputter Carbon/Gold Coater Systems, EDEN instruments, Alixan, France), then the examination of the gingival margins was conducted under the scanning electron microscope (JSM-6510LV SEM, JEOL Ltd, Tokyo, Japan) at 200x and 1000x magnifications with a voltage of 30 KV. Each restoration was considered as a testing unit specimen for this study. The entire length of the evaluated margin was traced to detect the formed marginal gaps by a blinded evaluator. The width of each detected gap was taken at different points and the mean width was calculated. On the basis of the mean width of the maximum marginal gaps, the margins were given scores as follows; score 0; no marginal gap formation, score 1; maximum marginal gap < 30 μm, score 2; maximum marginal gap > 30 μm [37].

### Statistical analysis

The extracted data were analyzed using the Statistical Package for the Social Sciences (IBM-SPSS, version 24, Armonk, NY, USA). After verifying the normal distribution of the data using Kolmogorov-Sminrov test (*p* > 0.05), Chi-square test was used to assess the categorical variables’ association at a significance level of *p* < 0.05.

## Results

Chi-square test indicated no statistically significant difference among all room temperature-stored groups either with margins located above C.E.J (*p* = 0.558) or below C.E.J (*p* = 0.807). As well, there was no statistically significant difference when comparing both marginal locations for Admira F (*p* = 0.286), Ceram X (*p* = 0.592), and Tetric N (*p* = 1.0). Score 2 was not observed for all room temperature-stored groups. Regarding groups stored in refrigerator, there was no statistically significant difference among all groups either with margins located above C.E.J (*p* = 1.0) or below C.E.J (*p* = 0.575). Moreover, no statistically significant difference was noted when comparing both marginal locations for Admira F (*p* = 0.1), Ceram X (*p* = 0.229), and Tetric N (*p* = 0.229). Score 0 was not recorded for all refrigerator-stored groups.

**Fig. 3:**
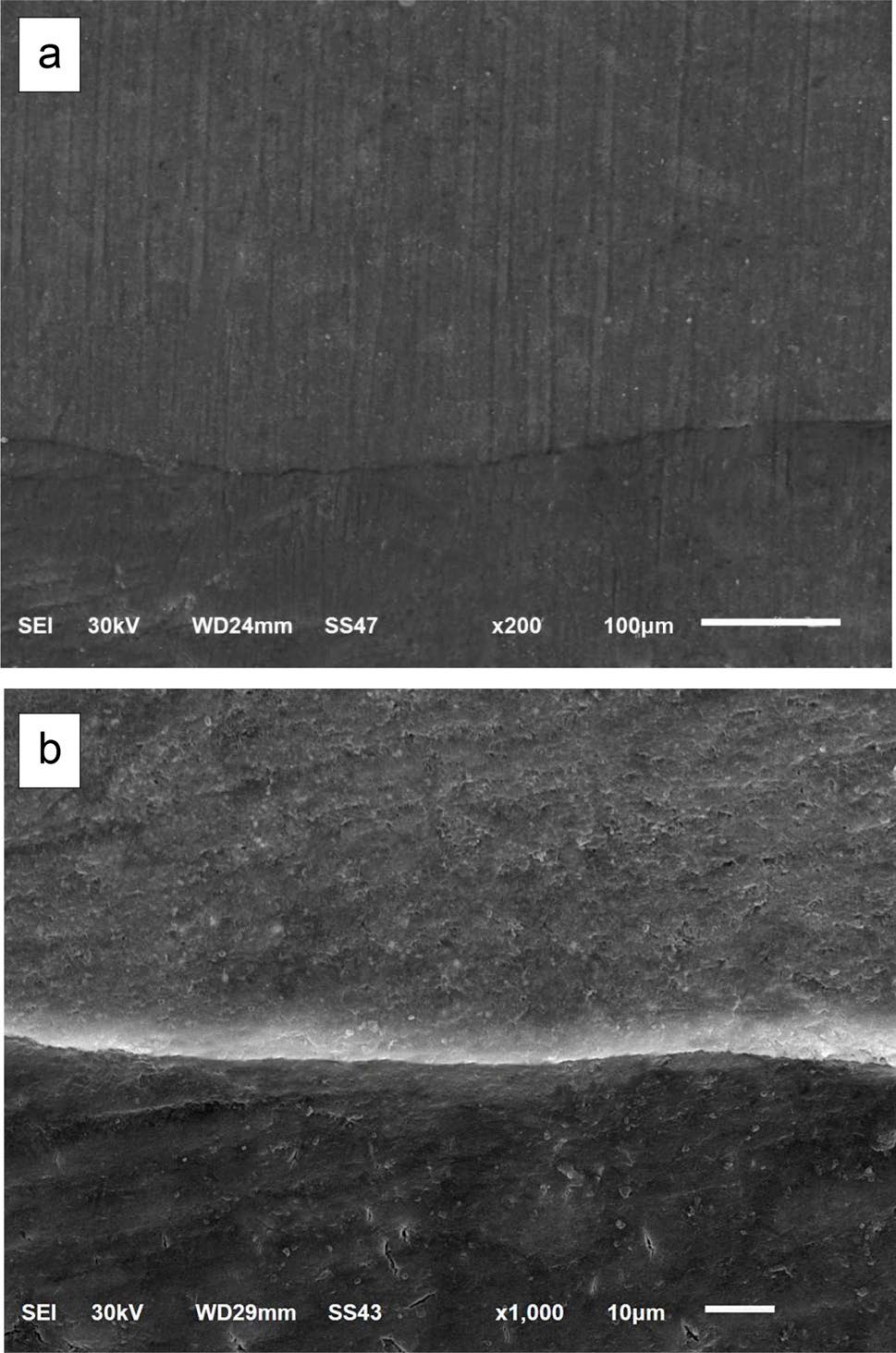
SEM photomicrograph of marginal adaptation with no marginal gap formation (score 0) at: **a**: 200x, **b**: 1000x

**Fig. 4:**
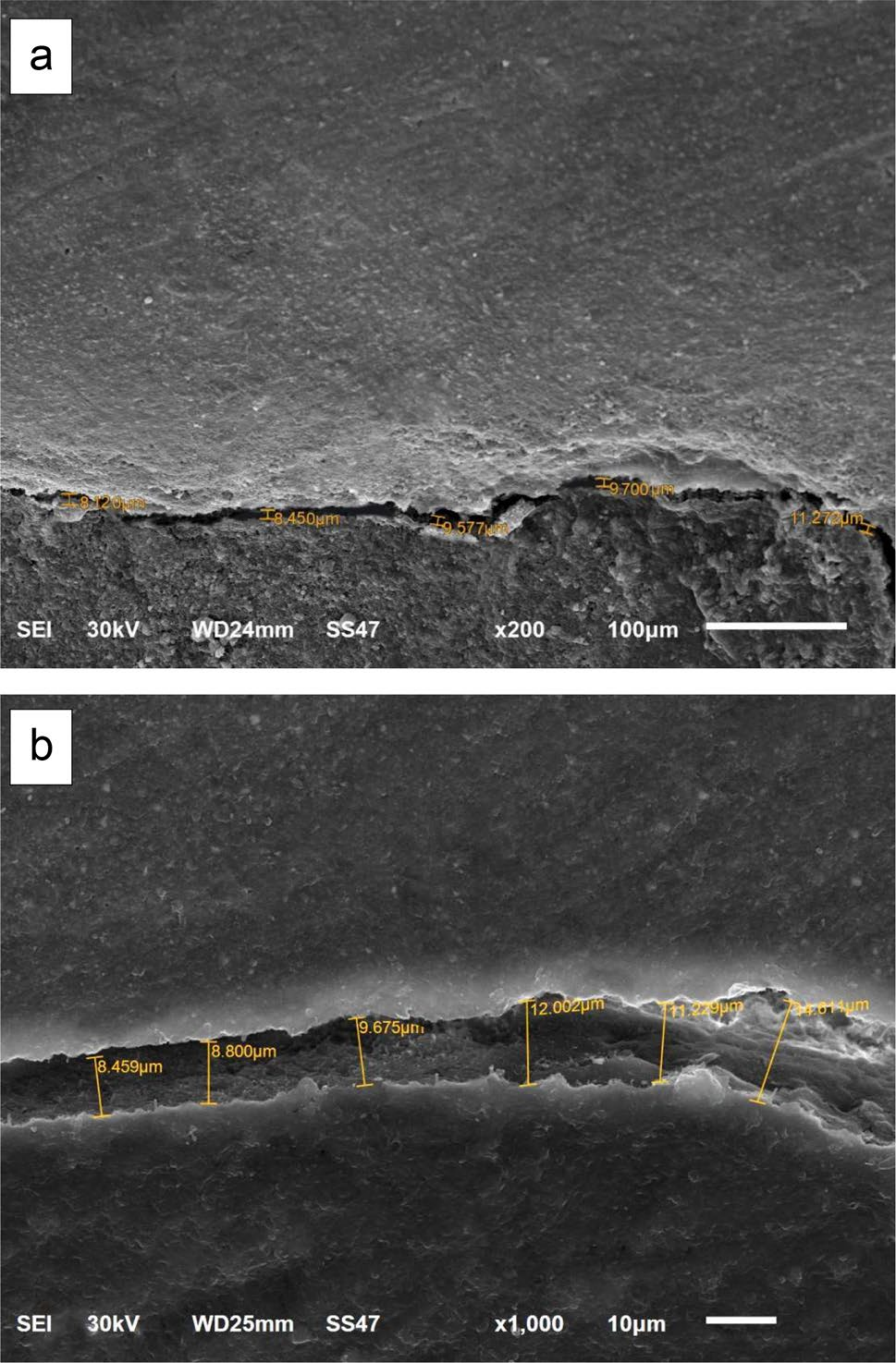
SEM photomicrograph of marginal adaptation with maximum marginal gap formation < 30 μm (score 1) at: **a**: 200x, **b**: 1000x

**Fig. 5:**
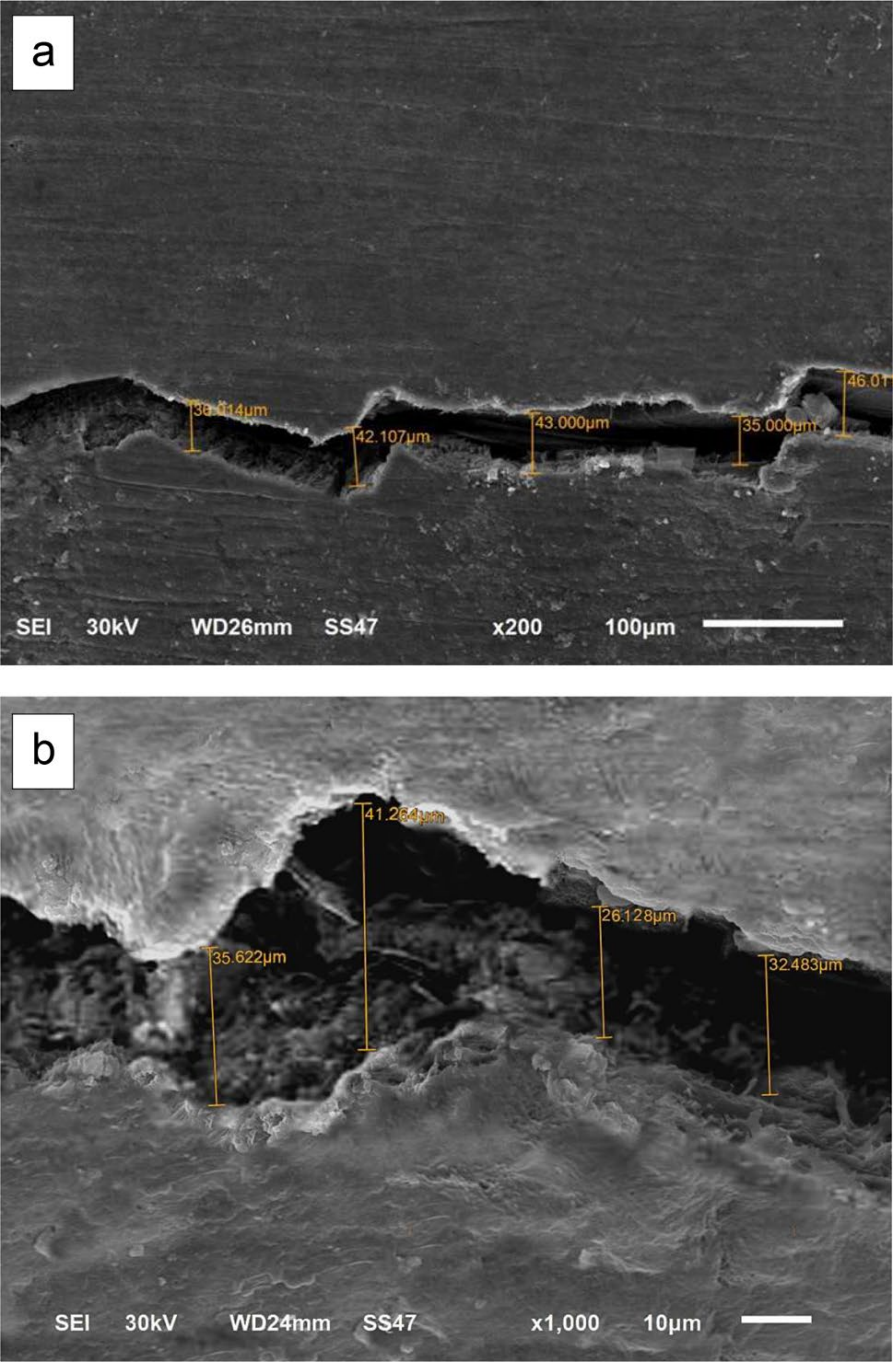
SEM photomicrograph of marginal adaptation with maximum marginal gap formation > 30 μm (score 2) at: **a**: 200x, **b**: 1000x

The comparison between the room temperature-stored groups and their counterparts of refrigerator-stored groups with margins located above C.E.J indicated a statistically significant difference for Admira F (*p* = 0.005), Ceram X (*p* = 0.018), and Tetric N (*p* = 0.05). However, the comparison between the room-temperature stored groups with margins located below C.E.J and their corresponding groups stored in refrigerator revealed no statistically significant difference for Admira F (*p* = 0.127), Ceram X (*p* = 0.213), and Tetric N (*p* = 0.111). All the results were expressed in the percent of specimens with its recorded score for each group (Table 2). The scores representing the maximum marginal gaps obtained by the scanning electron microscope (SEM) analyses of the epoxy replicas at 200x and 1000x magnifications are illustrated in Figs. 3, 4 and 5.

**Table 2:** Results of marginal adaptation scores for different groups

Material	Score	Margin above C.E.J		Margin below C.E.J	
		Room temperature	Refrigerator	Room temperature	Refrigerator
**Admira F**	Score 0	5 (71.4%)	0 (0%)	2 (28.6%)	0 (0%)
	Score 1	2 (28.6%)	7 (100%)	5 (71.4%)	7 (100%)
	Score 2	0 (0%)	0 (0%)	0 (0%)	0 (0%)
**Ceram X**	Score 0	4 (57.1%)	0 (0%)	2 (28.6%)	0 (0%)
	Score 1	3 (42.9%)	7 (100%)	5 (71.4%)	6 (85.7%)
	Score 2	0 (0%)	0 (0%)	0 (0%)	1 (14.3%)
**Tetric N**	Score 0	3 (42.9%)	0 (0%)	3 (42.9%)	0 (0%)
	Score 1	4 (57.1%)	7 (100%)	4 (57.1%)	6 (85.7%)
	Score 2	0 (0%)	0 (0%)	0 (0%)	1 (14.3%)
**score 0**: no marginal gap formation **score 1**: maximum marginal gap < 30 μm **score 2**: maximum marginal gap > 30 μm					

## Discussion

The present in-vitro study evaluated the effect of storage temperature of three commercially-available resin composite restorative materials with different matrix systems on their marginal adaptation in Class II restorations. As no sufficient information is available regarding the impact of storage temperature on Ormocer, two Ormocer based composites; a pure Ormocer-based and a methacrylate modified Ormocer-based composites were compared to a conventional methacrylate-based one.

Since inaccurate results may be revealed when a resin composite restorative material is tested with adhesive from another manufacturer [38], each tested material was used with its recommended adhesive system from the same manufacturer. To minimize the variability in the current study, the selected materials have approximately the same filler loading in order to focus on the relation between the adaptation of these materials to cavity margins and the difference in matrix formulation.

Clinical trials are considered the gold standard method to evaluate the clinical performance of different restorative materials. However, performing in-vitro studies is a significant method for testing new materials prior to their clinical application [39]. As well, providing the basis for recommendations on how clinicians should use resin composite restorative materials in their daily clinical practice [40].

The clinical effectiveness of resin composite restorative material can be generally judged by its adaptation to cavity details [41]. Moreover, the evidence of gap formation, irrespective of the width and length, impairs the marginal integrity and endangers on the clinical longevity [42]. It was reported also that the gingival dental zone is the area of maximum stress accumulation and the most challenging area to be restored [20]. Therefore, gingival marginal adaptation was chosen to be evaluated in the current study considering the difficulties facing the clinicians in restoring this particular area in Class II cavities [43]. The gingival margins of the prepared Class II proximal boxes were placed 1 mm above C.E.J at one side and 1 mm below C.E.J at the other side. This preparation design was chosen to evaluate the marginal adaptation of restorations to enamel and cementum regarding the difference in the matrix formulation as well, the storage temperature.

Standardized separated proximal Class II boxes were prepared using a straight fissure diamond instrument fixed in a high-speed headpiece which was attached to a specially designed jig and fixer to avoid bias and incorrect interpretation of the results [44]. The incremental technique in restorative procedures was used as it is considered the most appropriate method for placing resin composite restorative materials in order to decrease the deleterious effect of the polymerization shrinkage and associated stresses [45]. Also, the intensity of light curing device and the curing time were kept constant.

The gingival margins were evaluated under a scanning electron microscope (SEM) which is considered the most efficient, reliable, and highly discriminative assessment method for evaluating the marginal gaps and the continuity of tooth-restoration interface [37]. Direct visualization of the formed gaps is incompetent as a result of the vacuum procedure utilized during SEM analysis, which may cause cracks. By using a replica method, the formation of artificial gaps which are difficult to be differentiated from the contraction gaps can be avoided [20]. Moreover, completely dried teeth are required for direct SEM analysis, whereby existing marginal gaps may become wider as a result of dehydration. Therefore, it is preferable to observe positive epoxy resin replicas rather than the dehydrated tooth samples [34]. It was reported also that replica techniques are precise and appropriate for the analysis of the measurements of marginal gaps [46]. Additionally, the replica SEM method can be used to re-evaluate samples at different study levels without destroying original teeth which allow for a longer time-span monitoring [40].

The results of the current study showed that none of the tested groups could exhibit 100% gap-free margins irrespective of restorative material, margin location or storage temperature. This might be attributed to their matrix composition and filler content which hinder the proper flow to achieve adequate marginal adaptation. Moreover, the study results could be related to the difficulty of achieving optimum marginal adaptation in Class II restorations particularly when the cervical margins are placed below the C.E.J as reported by Krämer et al. [47]. This result was in agreement with Mahmoud et al. [38] who reported that no gap-free margins were achieved when the marginal adaptation of three different resin composite restorative materials was assessed.

The scores of marginal adaptation among the tested groups revealed insignificant difference at the different storage temperatures. A possible explanation for the formation of marginal gaps in composite restorations is very complex and multi-factorial process which could be related not only to the difference in materials’ formulations, but also to the interface stress during light photo-polymerization, quality of bonding approach, and difference in coefficient of thermal expansion [45].

Despite the insignificant difference among the scores of the tested groups, both Ormocer-based materials have shown slight better marginal adaptation at both margin locations as well as the different storage temperatures when compared to methacrylate-based composite. This might be attributed to the large sized molecule of Ormocer matrix and the polymerizable organic side chains bonded to the ceramic polyslioxane matrix that decreased the polymerization shrinkage stresses generated at the tooth-restoration interface. Hence, fewer gaps were formed and better marginal adaptation was achieved.

Additionally, the high viscosity of Bis-GMA monomer which is the main component of the matrix of methacrylate-based composite might be responsible of increasing polymerization shrinkage and associated stresses at the adhesive joint causing loss of marginal integrity. Consequently, high percentage of marginal gaps was formed as reported by Papadogiannis et al. [48] who stated that the shrinkage characteristics of resin composite restorative materials greatly influence their marginal adaptation. A study conducted by Sudhapalli et al. [49] showed a superior marginal sealing ability of Ormocer-based composite in comparison to methacrylate based composite; Tertic N-Ceram. On the contrary, Kournetas et al. [42] reported an inferior marginal adaptation for Ormocer-based composite when compared to a hybrid composite. Variations in findings might be related to the different evaluation methods used for assessing the marginal adaptation.

The outcome of the current study indicated insignificantly better marginal adaptation at enamel margins when compared to cementum margins regardless the restorative material or the storage temperature. This could be related to the role of the bonding approach used, as the universal adhesives were employed using selective etching technique to the enamel margins and self-etch technique for the cementum margins. This could justify such approximate results for both enamel and cementum marginal adaptation as reported by Stoleriu et al. [50] in terms of microleakage. The results of this study were in agreement with the results of Roggendorf et al. [51] who reported insignificantly superior adaptation to enamel margins as compared to non-enamel margins. In contrast with this finding, Takahashi et al. [52] reported that the adaptation of resin composite restorative materials at enamel margins was significantly preferable over cementum. This confliction might be related to the differences in the bonding techniques and the evaluation protocol.

In terms of storage temperature, the significantly worse marginal adaptation at enamel of refrigerator-stored groups when compared to their counterparts stored at room temperature might be attributed to the effect of low-temperature in increasing the materials’ viscosity which may have worsened the flow of refrigerated materials to the enamel margins and hindered resisting the adverse effects of polymerization shrinkage. As a consequence, the marginal adaptation to enamel may be impaired even with its low proportion of water and organic materials in comparison to dentin and cementum explaining the higher percentage of gap-formation at the enamel-restoration margin after refrigeration. Regarding marginal adaptation to cementum, a less distinct difference was observed between room temperature-stored groups and their counterparts of refrigerator-stored groups. This result was in agreement with Briso et al. [53] who stated that no significant difference was observed in marginal microleakage at the cementum-restoration interface in Class II cavities when the restorative systems were used immediately after refrigerator storage.

It must be noted that there are certain limitations in this current in-vitro study as some parameters in oral environment that affects marginal gap formation in posterior Class II resin composite restorations could not be duplicated in this study. Hence, further investigations are still needed to fully assess the effect of refrigeration on the properties of resin composite restorative materials.

## Conclusions

Within the limitations of the current study, it was concluded that all the tested resin composite restorative materials failed to achieve 100% gap-free margins in Class II restorations regardless the type of material used, the marginal location, or the storage temperature. Moreover, the refrigeration had a deleterious effect on marginal adaptation of resin composite restorative materials with different matrix systems when the gingival margins of the restorations were located in enamel.

## Data Availability

The datasets used and/or analysed during the current study are available from the corresponding author on reasonable request.

## Abbreviations

Ormocer: Organically modified ceramic
SEM: Scanning electron microscope
C.E.J: Cemento-enamel junction
LED: Light-emitting diode
HEMA: HydroxyethylMethacrylate
Bis-GMA: Bisphenol A-glycidyl methacrylate
Bis-EMA: Ethoxylated bisphenol-A dimethacrylate
TEGDMA: Triethylene glycol dimethacrylate
PENTA: Dipentaerythritol pentacrylate
MDP: Methacryloyloxydecyl dihydrogen phosphate
UDMA: Urethane dimethacrylate
MCAP: Methac carboxylic acid polymer
D3MA: Decandiolrylate dimethacrylate
Admira F: Admira Fusion
Ceram X: Ceram.X SphereTEC One
Tetric N: Tetric N Ceram
